# The Treatment of Fractures from a Common-Sense Point of View

**Published:** 1907-11-09

**Authors:** 


### Points in Surgery.

### THE TREATMENT OF FRACTURES FROM A COMMON-SENSE POINT OF VIEW.

### XII. FRACTURES OF THE SHAFT OF THE FEMUR.

When the shaft of the femur is fractured in its middle third, there is little or no displacement, except that due to eversion of the lower fragment, which is caused by the fact that the greater part of its weight lies to the outer side of its long axis. The upper fragment is too large and heavy to be flexed by the ilio-psoas muscle. It is therefore quite easy to treat such a fracture with a Liston's splint applied to the outer side of the limb and three short splints round the thigh. Liston's splint is better than Dessault's, which has attached to it a foot-piece; this is difficult to keep adequately applied to the foot. Extension must be applied by means of a weight passing over a pulley—about half a pound should be allowed for every year of the patient's age up to ten pounds. It is rarely necessary to use more than this. The weight is attached to a wooden stirrup, which is attached to the limb with transverse pieces of strapping. There are several small points to be observed in applying the stirrup to the limb. The extension must be applied to the lower end of the femur, and not to the tibia: if this precaution is not observed it is found that the patient is unable to walk at the end of convalescence, not because the bone is ununited, but because the ligaments in and surrounding the knee-joint have been weakened and stretched by the extension. It is well not to apply the strapping over bony prominences, particularly the malleoli. To avoid this the transverse pieces should stop short some distance above the ankle-joint, and the bar of the stirrup should be fairly long, so that the longitudinal pieces of strapping attached to it are carried out away from the malleoli. It will save the patient pain in the future if the leg is shaved before the strapping is applied: if this is not done its removal causes some inconvenience. The perineal band which was advised in text-books of surgery is nowadays not employed: if counter-extension is required it can be obtained by raising the foot of the bed and allowing the weight of the body to act. The extension will have done its work, and may be removed after three or four weeks. The splint should be left on for another fortnight, and the leg can then be put up in silicate or plaster of Paris. The former is preferable because it is lighter, and more easily kept clean. When the silicate bandages are being applied, they should not be rolled too tightly, because the silicate contracts as it hardens, and undue pressure on the limb may be caused. The patient may begin to get about on crutches after eight or nine weeks. In children this injury is best treated by means of a gallows-splint. This consists of a cross-bar of wood supported horizontally at a convenient height above the bed by upright bars which are fixed to the framework. Strapping as for extension by weight is attached to both legs, which are slung up to the crossbar, so that the sacrum is lifted from the bed. The weight of the body thus supplies the extension. This apparatus will be found very satisfactory. Its only disadvantage is that the strapping tends to slip, and the legs come out of the vertical line.

When the femur is fractured in the upper or in the lower third, the leg must be retained in the flexed position. In the first case the upper fragment is flexed and externally rotated by the ilio-psoas; and it is not advisable to extend it again and keep it in position with a pad, because the pressure required to do this would be injurious to the soft structures. In the second case the lower fragment is flexed at the knee by the two heads of the gastrocnemius muscle, which take their origin from the posterior surface of the condyles. In each case therefore the larger fragment, which can be controlled, must be brought into line with the smaller fragment, and this is done by putting the limb up in the flexed position. For this purpose Hodgen's splint is the apparatus *par excellence*, and its merits are now so universally acknowledged that it has practically replaced all other methods, and is used as a routine measure in hospital practice. It is most difficult to explain in words the method of applying this splint, and no one has any real knowledge of its working until he has had occasion to use it. Briefly, however, the method of its application is as follows: A stirrup for extension is applied to the limb with strapping. The splint itself consists of a thin wire frame-work, roughly of the same shape as the limb, which extends from Poupart's ligament to about nine inches beyond the sole of the foot, and is bent at a wide angle at a point corresponding to the knee. The limb is slung from this on broad pieces of house flannel, which are pinned to the sides of the splint. The stirrup is then fixed to the lower cross-bar of the framework. To each side of the framework two pieces of cord are attached, one near each end. These four pieces of cord meet at a common pulley, and from this a single cord leads to a pulley attached to a stout pole fixed to the foot of the bed. The height of this pulley is so arranged that when the cord is made tight its line of pull will be in the line of the femur. When all is ready the single cord is pulled upon until the leg is raised from the bed and is fastened to the pole. In practice the success of the method depends largely on small points, such as the degree of tightness of each strip of flannel and the proper adjustment of each of the four cords leading from the framework to the central pulley, and these can only be learnt by experience. But when it is once correctly adjusted there are few forms of retentive apparatus more satisfactory than a Hodgen's splint. The strapping generally holds for three or four weeks, after which time there is no need to continue the extension and the limb may then be put up in a silicate bandage. It is a specially useful splint for elderly patients or those with bronchitis, because they can be propped up without interfering with the position of the limb. Moreover, it allows nursing manipulations to be carried out with comparative ease.

